# Assessment of a New Silicon Carbide Tubular Honeycomb Membrane for Treatment of Olive Mill Wastewaters

**DOI:** 10.3390/membranes7010012

**Published:** 2017-02-27

**Authors:** Maria C. Fraga, Sandra Sanches, João G. Crespo, Vanessa J. Pereira

**Affiliations:** 1 REQUIMTE/LAQV, Department of Chemistry, Faculdade de Ciências e Tecnologia, Universidade Nova de Lisboa, Campus de Caparica, 2829-516 Caparica, Portugal; carmo.fraga@itqb.unl.pt; 2 Instituto de Biologia Experimental e Tecnológica, Apartado 12, 2781-901 Oeiras, Portugal; sandras@itqb.unl.pt

**Keywords:** Silicon carbide, membrane filtration, pilot scale, olive mill wastewaters, flux maintenance strategies, fouling prevention

## Abstract

Extremely high removals of total suspended solids and oil and grease were obtained when olive mill wastewaters were filtered using new silicon carbide tubular membranes. These new membranes were used at constant permeate flux to treat real olive mill wastewaters at pilot scale. The filtration conditions were evaluated and optimized in terms of the selection of the permeate flux and flux maintenance strategies employed—backpulsing and backwashing—in order to reduce fouling formation. The results obtained reveal that the combination of backpulses and backwashes helps to maintain the permeate flux, avoids transmembrane pressure increase and decreases the cake resistance. Moreover, membrane cleaning procedures were compared and the main agents responsible for fouling formation identified. Results also show that, under total recirculation, despite an increased concentration of pollutants in the feed stream, the quality of the permeate is maintained. Membrane filtration using silicon carbide membranes is an effective alternative to dissolved air flotation and can be applied efficiently to remove total suspended solids and oil and grease from olive mill wastewaters.

## 1. Introduction

Oily wastewaters are one of the main pollutants of the aquatic environment that, due to its hazardous nature, can cause serious environmental problems [1]. A large volume of these wastewaters is generated from various industrial processes, such as olive oil production, and needs to be treated before being discharged in the aquatic environment. The annual world production of olive oil, estimated in 2.5 × 10^6^ tons, is one of the most important agricultural activities in the Mediterranean countries, which are responsible for the production of 97% of the total world’s olive oil [2,3]. However, such a high production of olive oil also results in an extremely high production of wastewaters characterized by a high concentration of total suspended solids and organic compounds (polysaccharides, phenols, polyalcohols, proteins, organic acids and oil) [4,5,6]. The physical and chemical composition of olive mill wastewaters depend on several factors such as olive extraction processes and olive maturation as well as climatic and agronomic conditions [7,8].

Due to the presence of phytotoxic and antibacterial phenolic substances, these wastewaters are often resistant to biological degradation [8,9]. Traditional treatment methods, including skimming, coagulation, flocculation, sedimentation and flotation, present disadvantages such as low efficiency in the treatment of stable emulsions, high sludge production, high operation costs or need to add chemicals. In this context, membrane technology has become a significant separation process over the last years [10,11,12,13,14], being efficient in treating stable emulsions, allowing high quality of permeate produced (the variation in feed water quality will have a minimal impact on permeate quality) and generating a small volume of waste requiring further treatment [1,15]. Moreover, membranes require small implementation areas and the use of chemicals is avoided. Regarding the costs, membrane processes present low investment and maintenance costs, high efficiency and low energy consumption [16]. The use of membrane technology can therefore be compared to conventional processes for different wastewater applications.

Membrane fouling and its consequent flux decline (when the filtration process is performed under constant transmembrane pressure) or transmembrane pressure increase (when the filtration process is performed under constant permeate flux conditions) is the main drawback of pressure-driven membrane separation processes. Even though most studies in the literature operate at constant transmembrane pressure, most industrial water purification membrane installations operate at constant flux [17]. Working at constant permeate flux seems to be a valid strategy to reduce the fouling occurrence rather than working at constant-pressure operation [18]. Miller et al. [19] compared membrane fouling in the filtration of oily wastewater with polysulfone membranes with 20 kDa molecular weight cut off. They observed that, working below a specific threshold flux, a constant flux operation minimizes fouling appearance and membrane resistance.

It is important to define the permeate flux at which fouling is first observed for a given feed concentration to optimize the membrane process and minimize fouling [20,21].

Besides defining an optimum operating permeate flux, different cleaning systems can be applied as flux maintenance strategies such as backpulse (BP), backwash (BW), chemically enhanced backwash (CEB) and cleaning in place (CIP) [21]. The effect of backpulses and backwashes in the microfiltration of oil-in-water emulsions with ceramic membranes was already studied and reported. Results show that these strategies are efficient in fouling prevention without decreasing the oil rejection [22,23,24]. From an economic point of view, a study comparing the microfiltration of emulsified crude oil with and without backpulses revealed that the process without backpulses is not economically viable when compared to conventional treatment methods. However, the same operation with regular backpulses resulted in lower costs of treated water when compared with conventional methods [25].

The use of ceramic membranes recently increased, mainly for application in industrial processes [26]. Due to their advantadges compared with polymeric membranes—including better thermal stability, mechanical resistance and chemical resistance—ceramic membranes can be applied in extremely aggressive environmental conditions [27]. These properties allow for better control of membrane fouling since higher pressures can be employed in backwashes and cleanings can be performed with stronger chemicals, while extending the membrane lifetime [28]. Satisfactory results in the treatment of oily wastewaters were reported when microfiltration ceramic membranes were used [22,23,29]. γ-alumina is often used as a support material for ultrafiltration membranes due to its smooth surface, in contrast to other materials, and since it is fairly inert. On the other hand, it can be easily deposited in macroporous supports. Nevertheless, γ-alumina do not present high enough chemical or mechanical stability when subject to severe conditions. A promising material for ceramic membranes is silicon carbide (SiC) since it presents better resistance to chemicals, and thus presents advantages when strong and repeated cleanings are required [30,31]. Moreover, when compared with polymeric and other ceramic membranes such as titania or zirconia, silicon carbide membranes present higher hydrophilicity and lower fouling tendency [32] and thus allow higher permeate fluxes in wastewater treatment.

In the present work, a new silicon carbide tubular ceramic membrane [33], with a single retentive layer on top of the substrate was tested, for the first time, to treat real olive mill wastewaters at pilot scale. This work focused in the optimization of constant flux filtration conditions. Backpulses and backwashes were studied in order to reduce the fouling formation and consequently avoid transmembrane pressure increase. The filtration studies were performed under total recirculation conditions and a final concentration test was conducted under optimized conditions. Different cleaning protocols were also tested in order to optimize the chemical cleaning of the membrane.

## 2. Material and methods

### 2.1. Characterization of Pilot Scale Unit, Membranes and Wastewater Matrices

**Figure 1:**
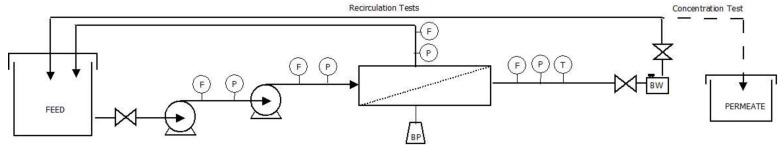
Scheme of the pilot filtration unit with cleaning devices (BP—Backpulse and BW—Backwash) used to treat the real olive mill wastewater in different operation modes (recirculation and concentration tests).

Olive mill wastewater, collected after the sedimentation process at a real wastewater treatment plant, was processed in a pilot scale membrane filtration unit (LabBrain unit, depicted in Figure 1), with cleaning devices (backpulse and backwash) incorporated and automatic data acquisition of transmembrane pressure (TMP) and permeate flux.

The characteristics of the new tubular honeycomb silicon carbide membranes (produced by LiqTech, Ballerup, Denmark) used in this study are detailed in Table 1. These membranes were developed in the scope of a joint project, previously characterized in terms of morphology, chemical surface composition and effectiveness to treat a different matrix (sunflower oil wastewater) [33]. The manufacturing process of these new membranes allows time and economic savings when compared with commercially available membranes with two layers (a top and an intermediate layer). It is extremely interesting to observe that, in spite of the relatively low membrane porosity (Table 1), this membrane presents a high hydraulic permeability, possibly as a result of its high hydrophilic character. In this study, contact angle measurements were performed (KSV Instruments LTD, CAM 100, Helsinki, Finland, with the software KSV CAM2008) to further characterize the new membrane (Table 1). However, a stable contact angle could not be determined because the coating of the membrane is extremely hydrophilic and the water drop was readily absorbed by the membrane. For nine different zones of the membrane, the average first contact angle value was determined. In addition, since the water drop was readily absorbed by the membrane, the contact angle decrease was also followed over time in the nine different zones. The measurements were performed with frame intervals of 100 ms. The contact angle values measured over time adjusted to a linear regression and the average of all the slopes obtained (velocity of decreasing contact angle) are also presented in Table 1.

The pilot scale installation is built in stainless steel (AISI-304) and all components in contact with liquids are stainless steel AISI-316 with Teflon-coated components or Viton/EPDM/Nitrile sealing gaskets. The LabBrain Unit (LiqTech, Ballerup, Denmark) is equipped with a feed pump (Grundfos CRN 1-3, Bjerringbro, Denmark) and a recirculation pump which generates the crossflow (Grundfos CRN 3-4, Bjerringbro, Denmark). The unit can be operated both under crossflow and semi-dead-end mode; in this work the filtration was performed in crossflow mode. The pressure and flow rate inside the system are controlled by adjusting the position of the regulating valves and the pump speed. All data from pressure transmitters, flow transmitters, temperature transmitters, pump settings and valve positions are stored in the internal memory of the unit.

In addition, the unit is equipped with a Back Pulse Hammer (BPH). The BPH system is a pulse generator delivering high frequency “block” pulses from the permeate side, back through the membrane in order to keep the membrane clean and free of foulants. Backwash controlled by compressed air is also integrated in the unit. Both backpulses and backwashes can be performed manually or automatically. Various terminologies are applied in the literature regarding cleaning devices. In this work, the term “backpulse” will refer to very short air pulses generated from the permeate side, whose function is to loosen foulants, which are then removed by the crossflow. The term “backwash” will refer to the reversion of the permeate flow by means of a pump. In this case, the foulants on the membrane surface are washed away by the reversed permeate flow and removed by the crossflow.

Table 2 presents an average of the parameters analysed (total solids—Standard Method 2540B [34], total suspended solids—Standard Method 2540D [34], chemical oxygen demand (COD)—Standard Method 5220 [34], total organic carbon (TOC)—Standard Method 5310B and oil and grease—Standard Method 5520C [34]) of the wastewater samples collected in six different sampling events corresponding to the six tests performed, showing that concentration of the five parameters analysed are highly superior to the limits imposed by the legislation for direct discharge in watercourses.

### 2.2. Membrane Filtration Tests

#### 2.2.1. Determination of Optimal Permeate Flux Conditions 

To define the best operating flux that minimizes fouling for further application in long-term filtration assays, a preliminary study was carried out using the pretreated wastewater samples by assessing transmembrane pressure (TMP) variations under different constant permeate flowrates set during five-minute intervals. The selected permeate flux to conduct the experiments was the one at which a lower TMP variation was observed.

#### 2.2.2. Total Recirculation Tests

Table 3 summarizes the operating conditions set in each filtration test. Four 24 h assays (tests 1–4) were conducted in total recirculation mode with a crossflow velocity set at 2 m·s^−1^ and the previously determined optimum permeate flux value.

During the 24 h long assays, the variation of transmembrane pressure was followed and the effect of backpulse (every 10 min) and backwash (every 2 and 1 h), employed as flux maintenance strategies, were studied. The permeate flux and pressure data acquisition in the LabBrain unit was automatically stored. A first test without cleaning strategies was performed (test 1). In order to study the effect of backpulses, a second test was carried out employing backpulses every 10 min (test 2). In tests 3 and 4, besides backpulses every 10 min, backwashes were also employed every two hours (test 3) and every hour (test 4) to study the effect of the combined flux maintenance strategies. These intervals were set based on experience of the manufacturer with other emulsified wastewaters and several assays performed with the unit and different wastewater qualities (data not shown).

The effectiveness of the membrane filtration assays was evaluated by monitoring TMP variation over time at the different imposed permeate fluxes and calculating the consequent membrane resistance levels as well as by determining the percent rejection and adsorption/deposition to the silicon carbide membranes of different water quality parameters (total solids, total suspended solids, chemical oxygen demand, total organic carbon and oil and grease). Samples were stored at 4 °C until analysis.

#### 2.2.3. Optimization of Membrane Cleaning

In order to find out the best cleaning strategy to recover the permeability of the membrane, the effect of using different cleaning solutions and temperatures (25 °C and 65 ± 5 °C) was studied. Solutions of NaOH 4% (w/v) and citric acid 2% (w/v) were tested. The recovery of the permeability achieved in each cleaning step was determined to understand the efficiency of each cleaning. The permeability of the membrane was considered to be restored when 90% of its hydraulic permeability was recovered. A mass balance was performed to compare the concentrations of different water quality parameters detected in the cleaning solutions with the levels of adsorption calculated in the filtration assays, to gain further knowledge about the efficiency of the different cleaning steps.

#### 2.2.4. Concentration Test

In order to test conditions that best simulate the real conditions, a final concentration test was performed in the same unit. A quantity of 58 L of a pretreated olive mill wastewater was filtered with total recirculation of the retentate and total recovery of the permeate. Several samples were taken during the assay in order to evaluate the effectiveness of the membrane filtration in terms of the target parameters.

The starting conditions of the concentration test were set according to the optimum conditions selected in the total recirculation tests. Nevertheless, and due to a better quality of the wastewater received, no significant variation of TMP was observed after one hour of filtration; therefore, the permeate flux was incremented to 100 L·m^−2^·h^−1^ to increase the water production. During the entire filtration assay, backpulses (every 10 min) and backwashes (every hour) were applied. After the 7 h assay, a volume reduction factor of 5.2 was achieved.

## 3. Results and Discussion

### 3.1. Membrane Filtration Tests

#### 3.1.1. Determination of Controlled Permeate Flux Operating Conditions

In order to determine the optimum permeate flux for the 24 h filtration assays, different controlled permeate fluxes were set for 5 min and the corresponding TMP values recorded. The chosen flux was the one for which a lower increase in TMP was observed, in order to ensure a minimal fouling under long-term operation.

**Figure 2:**
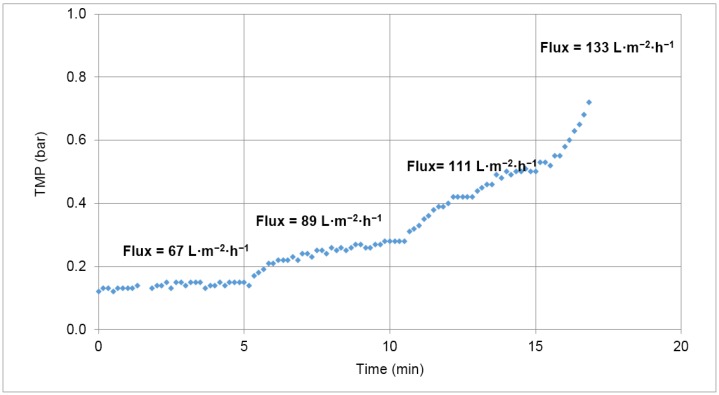
Variation of transmembrane pressure (TMP) with increase of controlled permeate flux.

Figure 2 shows the TMP increasing with the permeate flux variation. Due to limitations of the system used, it was not possible to test fluxes lower than 67 L·m^−2^·h^−1^. Although some fouling was observed in each step, resulting in TMP increase in all of them, the value of 67 L·m^−2^·h^−1^ was the chosen permeate flux to initiate the tests since at this permeate flux the lowest TMP increase was observed.

#### 3.1.2. Total Recirculation Tests

**Figure 3:**
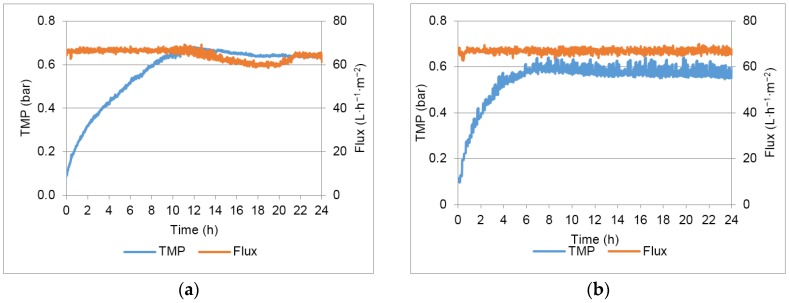

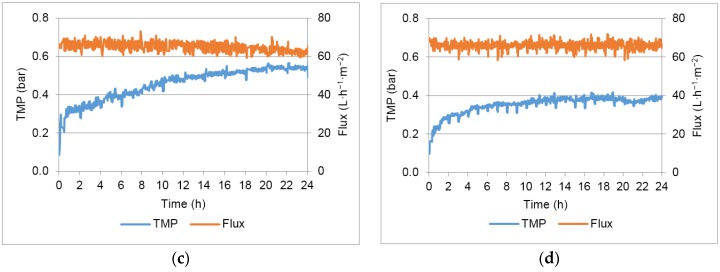
TMP and flux profiles obtained in the different assays: (**a**) test 1; (**b**) test 2; (**c**) test 3; (**d**) test 4.

Figure 3 shows the TMP variation in the different 24 h assays conducted with the chosen permeate flux (67 L·m^−2^·h^−1^) and different flux maintenance strategies (detailed in Table 3).

The effectiveness of the different flux maintenance strategies was calculated using Equation (1), where ∆*TMP_T1_* is the total variation of the TMP in the 24 h of test without flux maintenance strategy (test 1) and ∆*TMP_test_* refers to the variation of TMP in each test.

(1) $$\eta (\%)=100\times \frac{\left({\Delta TMP}_{T1}-{\Delta TMP}_{test}\right)}{{\Delta TMP}_{T1}}$$

In tests 2 and 3 a positive effect of backpulse (test 2) and backpulse combined with backwashing each 2 h (test 3) was observed compared with test 1 (no flux maintenance strategies and a TMP variation of 0.53 bar in the 24 h of assay). A transmembrane pressure variation of 0.48 and 0.43 bar in the 24 h was observed in tests 2 and 3, respectively. When the filtration assay was performed with backpulses each 10 min and backwashing each hour (test 4), a transmembrane pressure variation of 0.28 bar was obtained, nearly half the variation of transmembrane pressure observed when no flux maintenance strategies were applied, indicating that these strategies are rather efficient for fouling mitigation.

The higher effectiveness (𝝶) value presented in Table 4 indicates a lower fouling potential when the combined flux maintenance strategies were applied in test 4. The trend observed was expected: as the flux maintenance strategies are intensified, the effectiveness increases.

After test 4, in order to improve the permeate production, a new test was performed increasing 50% of the controlled permeate flux (100 L·m^−2^·h^−1^). However, under these conditions, a flux decrease of 55% was observed in the 24 hour assay which indicated that, for the oily wastewater tested, it was not possible to maintain this higher flux even when the flux maintenance systems are applied.

Using the optimal conditions (test 4), that allowed operation at a lower transmembrane pressure variation, a fouling rate was calculated using the TMP values recorded between 10 and 24 h. The fouling rate obtained (6 × 10^−4^ bar/h) was used to estimate the time needed to achieve 0.64 bar (the TMP obtained without flux maintenance strategies). The result obtained estimates an operation of 19 days using the optimal conditions proposed and shows that a long term continuous operation (without the need to stop the process and perform chemical cleanings) can be expected using these conditions.

Table 5 summarizes the percent rejection and adsorption/deposition related with the different parameters—total solids, total suspended solids, chemical oxygen demand, total organic carbon and oil and grease. The apparent rejection of the different parameters was calculated according to the following equation:

(2) $$\%Apparent\;rejection=\frac{C_{f}-C_{p}}{C_{f}}\times 100$$

where *C_f_* is the concentration of the different parameters in the feed water, *C_p_* is the concentration of the different parameters in the permeate stream (Table 6). The percent adsorption or deposition of the different parameters in the total recirculation tests was calculated according to Equation (3).

(3) $$\%Adsorption/Deposition=\frac{C_{f0}\times V_{f0}-C_{f24}\times V_{f24}}{C_{f0}\times V_{f0}}\times 100$$

where *C_f_*_0_ and *C_f_*_24_ are the concentrations of the parameters in the feed tank at t = 0 and 24, respectively and *V_f_*_0_ and *V_f_*_24_ are the volumes of feed at t = 0 and 24 h, respectively.

Extremely high percent removals of total suspended solids (>99%) and oil and grease (>97%) were observed in tests 1–4. Table 6 shows that membrane filtration ensures removals of these parameters until values lower than the legislation discharge limits. Removal of oil and grease is significantly due to adsorption/deposition on the membrane surface. The high adsorption/deposition of oil and grease was minimized by 48% using the optimized flux maintenance strategy (test 4).

Yang et al. [35] prepared a ZrO_2_/α-Al_2_O_3_ microfiltration membrane to treat oil-in-water emulsions, obtaining removals higher than 99% of oil. However, the hydraulic permeability of the microfiltration membranes were much lower than the hydraulic permeability of the silicon carbide membranes used in this work. Cui et al. [23] also reported removals higher than 99% of oil when using NaA/α-Al_2_O_3_ membranes to treat oil-in-water emulsions. In this case, the permeate fluxes were only 5 and 18 L·m^−2^·h^−1^, with a filtration time of 600 min. Regarding polymeric membranes, good oil and grease removals were also reported but with higher transmembrane pressures [36,37]. Ochando-Pulido et al. [38] achieved extremely high removals of total suspended solids from olive mill wastewaters by an ultrafiltration process using polymeric membranes but, once again, with fluxes not higher than 10 L·m^−2^·h^−1^. The silicon carbide membranes tested in this study ensure extremely high removals of oil and grease and total suspended solids allowing high permeate fluxes with low transmembrane pressure.

Lower removals of total solids, chemical oxygen demand and total organic carbon were observed, achieving up to 69% of chemical oxygen demand rejection in test 4 and 68% of total organic carbon rejection in test 3. Using the optimized conditions, higher values of rejection of all the tested parameters, except TOC, were obtained by ultrafiltration (this study, Table 5, test 4) compared to the removal values obtained by dissolved air flotation reported in a previous study (Total solids: 27%, total suspended solids: 98%, COD: 67%, TOC: 72%, Oil and grease: 77%; [39]). Ultrafiltration can therefore be applied instead of flotation for the treatment of olive mill wastewaters. COD removal was not enough to achieve values under the limit legislated. However, good percent removals were achieved when compared with other studied membrane processes: Coskun et al. [40] achieved the same range of removals combining ultrafiltration and nanofiltration to treat olive mill wastewaters. A previous study [41] obtained a maximum removal of 15% of COD from an olive mill wastewater using a regenerated cellulose membrane in dead-end configuration. The results obtained in this study are extremely promising since tests were performed using robust ceramic membranes and in conditions closer to reality in terms of flow dynamics. The membranes tested can achieve good removals with only one membrane step, maintaining a high permeate flux, during prolonged operation periods, with a low transmembrane pressure increase.

Higher percent adsorption/deposition values were reported in the assay without flux maintenance strategies (test 1) compared to the assays conducted with backpulse (test 2) and the tests conducted with backpulse and backwash (tests 3 and 4). These results were expected since backpulse and backwash are used to release the fouling components from the membrane surface.

The total membrane resistance (*R_t_*), corresponding to the sum of the membrane resistance (*R_m_*) and the resistance due to fouling (*R_f_*) in tests 1 to 4 was calculated at t = 24 h using Equation (4):

(4) $$R_{t}=R_{m}+R_{f}=\frac{TMP}{{µ}_{t}\times J}$$

where *TMP* refers to the transmembrane pressure, *J* to the permeate flux and µ_t_ to the fluid viscosity corrected to the working temperature, according to Equation (5) [42]:

(5) $${µ}_{t}=1.784-(0.0575\times T)+(0.0011\times T^{2})-(10^{-5}\times T^{3})$$

The value of the membrane resistance was determined (*R_m_* = 1.58 × 10^11^ m^−1^) using the value of the hydraulic permeability determined during clean water flux measurements. In order to analyse the effect of the cleaning strategies in the fouling formation, the values of resistance due to fouling of tests 1–4 were calculated and results clearly show the effect of backpulse and backwash strategies in the total resistance of the membrane. In test 1, conducted without flux maintenance strategies, the resistance of the membrane due to fouling at the end of the test was 4.14 × 10^12^ m^−1^. The use of backpulses each 10 min resulted in a decrease of the resistance of the membrane due to fouling to 3.75 × 10^12^ m^−1^. With backwashes each two hours in addition to the backpulses (*R_f_* = 3.54 × 10^12^ m^−1^) the difference was minor but when backwashes were performed each hour an accentuated decrease in membrane resistance due to fouling was observed (2.29 × 10^12^ m^−1)^. The considerable reduction in the resistance due to fouling, observed in test 4, may be interpreted taking into consideration the results presented in Table 5. The only parameter that could justify this difference taking into account the deposition/adsorption results is oil and grease. Therefore, it can be concluded that the reduction of the fouling resistance can be due to an effective release of oil and grease from the surface of the membrane when backpulses each 10 min are combined with backwashes every hour. The conditions employed in test 4 were therefore applied in a final concentration study, that better simulates real filtration conditions, conducted with total recirculation of the retentate and total recovery of the permeate. 

#### 3.1.3. Optimization of Membrane Cleaning

In order to optimize the cleaning protocol of the membrane, different cleaning solutions were tested and analysed in terms of total suspended solids and oil and grease—the contaminants considered to be the most important in fouling formation. In all tests, the first cleaning step was a rinsing step with hot water (60 ± 5 °C). Alkaline and acid solutions were tested after the rinsing step, and the effect of the temperature of the cleaning solutions was studied.

The first approach included the use of a 4% NaOH solution, recommended by the membrane manufacturer since it has a low cost, is easily available and can efficiently remove the oil and grease adsorbed on the surface of the membrane [43].

**Figure 4:**
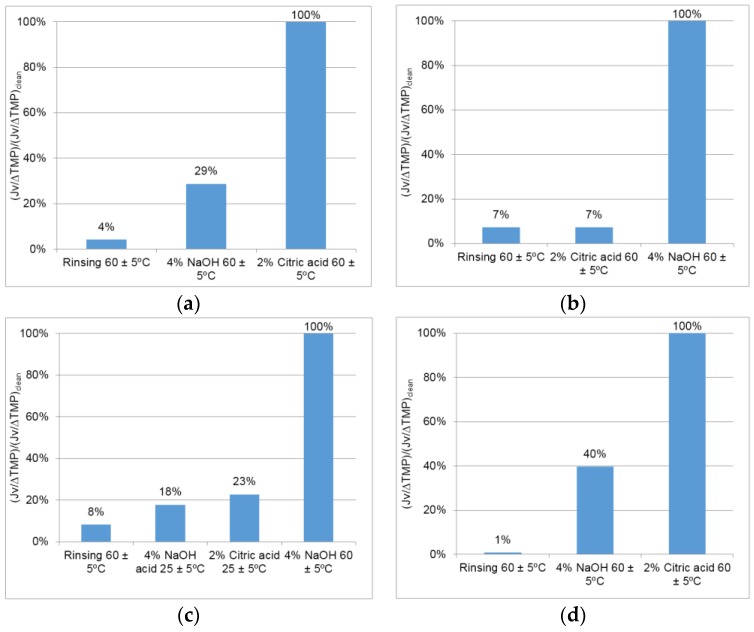
Percent recovery of the permeate flux per transmembrane pressure applied (*J_v_*/Δ*TMP*)/( *J_v_*/Δ*TMP*)_clean_ with different cleaning protocols performed after the membrane filtration assays: (**a**) test 1; (**b**) test 2; (**c**) test 3; (**d**) test 4.

Figure 4a shows the permeability of the membrane recovered after each cleaning step in test 1. Results show that rinsing and using NaOH at controlled temperature (60 ± 5 °C) was not enough to recover the permeability of the membrane. A solution of 2% citric acid was therefore employed. The results obtained show that the permeability was totally restored. It was thus concluded that the use of an acid solution may also be important to recover the permeability of the membrane with this wastewater.

After test 2 (Figure 4b), the strategy to clean the membrane was therefore the use of both acid and alkaline solutions at 60 ± 5 °C after an initial rinsing step. In this protocol, the sequence of steps was inverted, with the acid cleaning performed before the alkaline cleaning. The acid solution by itself was not enough to recover the permeability and only 6.5% of the adsorbed total suspended solids were recovered in this step. Even though total suspended solids and oil and grease were not detected after the acid cleaning step, a quick recovery of permeability was obtained using the consecutive acid and basic cleaning agents.

To understand if the use of a high temperature was really needed, cleaning after test 3 was performed with acid and alkaline solutions at room temperature—25 °C after rinsing with hot water (Figure 7c). The cleanings performed at 25 °C were not enough to restore the membrane permeability. After cleaning with NaOH at 60 ± 5 °C, the permeability was totally restored, so it was concluded that it is necessary to increase the temperature at least in one step of the chemical cleaning. A total of 100% of the suspended solids and 23% of the oil and grease adsorbed on the membrane were detected after analysing the cleaning solutions. Again, rinsing with hot water proved to be the most important step in the removal of total suspended solids (57%) and oil and grease (100%); 4% of the adsorbed total suspended solids was detected in the acid solution.

To study if the sequence of the chemical cleanings was important, in test 4 (Figure 4d) this procedure was performed after rinsing with an alkaline cleaning followed by an acid cleaning both at 60 ± 5 °C. It was observed that the permeability was totally restored after a sequence of basic and acid cleaning steps, indicating that the sequence does not seem to be important (compared with Figure 7b). Nevertheless, alternating alkaline and acid cleanings seems to be important in addition to the temperature: results indicate that the first chemical cleaning contributes to the destructuring of the existent fouling facilitating the subsequent cleaning. Furthermore, 75% of the total suspended solids removed using this cleaning protocol were recovered in the rinsing step, 21% in the alkaline cleaning and 4% in the acid cleaning. In sum, 82% of the total suspended solids adsorbed on the membrane surface were recovered in the cleaning procedure. All the adsorbed oil and grease were recovered in the rinsing step. Since the permeate flux was totally restored after the proposed cleaning procedure, the results indicate that total suspended solids, oil and grease and inorganic matter are important agents involved in fouling formation during the filtration of these wastewaters.

#### 3.1.4. Concentration Test

**Figure 5:**
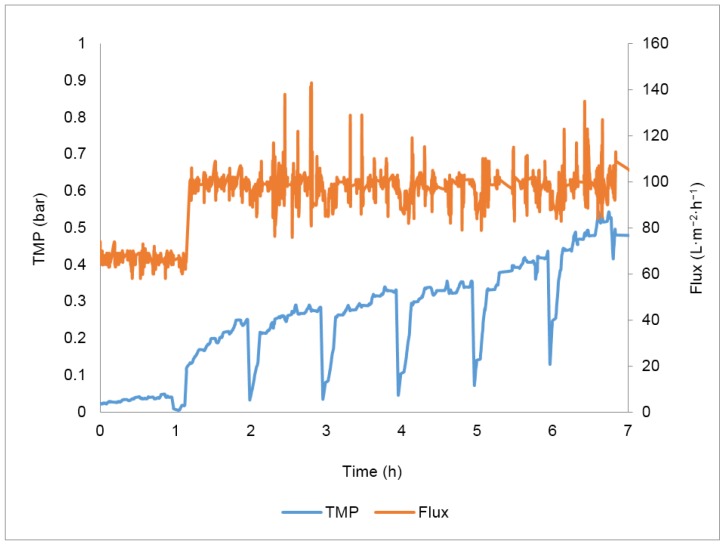
Transmembrane pressure (TMP) and permeate flux profiles obtained in the concentration test.

Figure 5 shows the TMP variation during the concentration test. During the first hour, the flux was set at 67 L·m^−2^·h^−1^ and backpulses every 10 min were performed in addition to backwashes every hour; the optimized conditions were determined in the total recirculation tests. The transmembrane pressure variation during the first hour was only 0.02 bar, very low compared to 0.15 bar variation obtained in the same period in the total recirculation assay—Figure 3d. In order to increase the process efficiency, the controlled permeate flux was therefore increased 50% in relation to the initial permeate flux, to 100 L·m^−2^·h^−1^, while keeping the flux maintenance strategies previously optimized.

The lower transmembrane pressure variation in this assay was due to a better quality of the large volume of oily wastewater received for the concentration study (Table 7), that was much less concentrated in terms of the water quality parameters analysed.

(6) $$Concentration\;factor=\frac{Volume\;feed}{Volume\;feed-Volume\;permeate}$$

In these conditions, a final concentration factor (Equation (6)) of 5.2 was achieved, corresponding to a permeate recovery of 81%.

**Figure 6:**
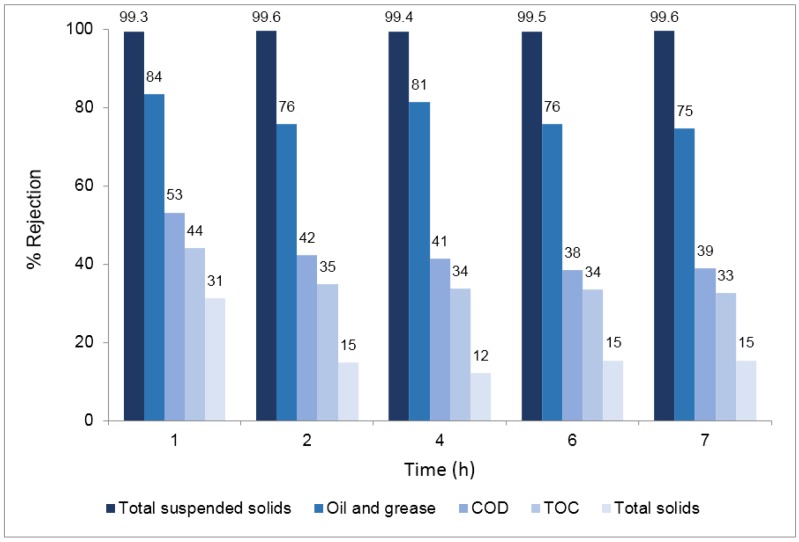
Percent rejection of total suspended solids, oil and grease, chemical oxygen demand (COD), total organic carbon (TOC) and total solids—Concentration test.

Figure 6 presents the percent rejection of the different parameters obtained in samples collected during the concentration assay.

Results show that the rejections of the different parameters monitored were maintained during the 7 h concentration study, evidencing that the quality of the permeate over time was not deteriorated despite the increasing concentration of the different components in the feed wastewater due to the total recirculation of the retentate. The results obtained in terms of rejection were consistent with the results previously obtained in the 24 h total recirculation test. The silicon carbide membranes used ensure high removals of total suspended solids and oil and grease. The value of membrane resistance at working temperature due to fouling at the end of this test was 2.31 × 10^12^ m^−1^.

Huang et al. [44] adapted the Hermia’s model [45] to describe fouling mechanisms in membrane processes performed at constant TMP and developed a similar one for membrane processes conducted at controlled permeate flux. This model was applied to the results obtained in the concentration test in order to identify the different fouling mechanisms involved. The results obtained indicate that the main fouling mechanism involved in this process is cake formation, since it presents the best coefficient of determination (0.92). This result is in accordance with other published studies, where the fouling formation during the ultrafiltration of oily wastewaters is mainly attributed to cake formation [46,47]. This fouling mechanism is attributed to the deposit of large molecules on the membrane surface. Results are thus in accordance with the assumption that total suspended solids and oil and grease are important parameters in fouling formation [48].

**Figure 7:**
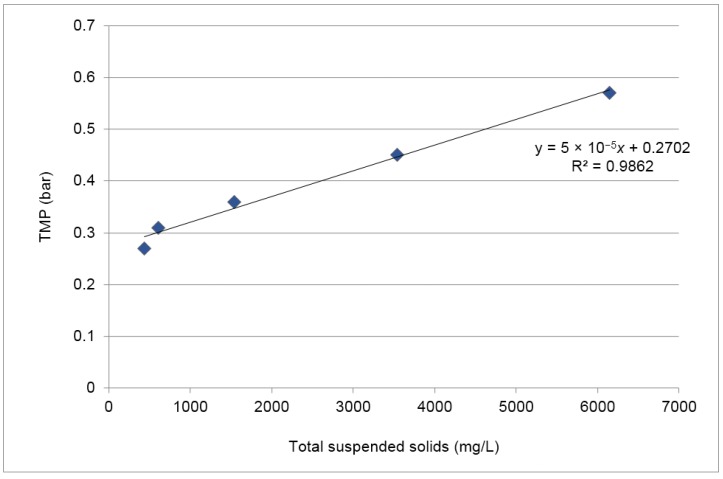
Impact of total suspended solids present in the feed in TMP—Concentration test.

Figure 7 relates the maximum TMP achieved before backwashes with the concentration of total suspended solids present in the feed at the same time. A linear regression with a coefficient of determination (R^2^) of 0.99 was obtained, indicating a strong relationship between these two variables and confirming the influence of the concentration of total suspended solids present in the feed in the cake formation. 

The optimized cleaning procedure was applied after the concentration test to restore the membrane permeability. In this case, after the alkaline step, the permeability was totally restored and the acid cleaning step was therefore not needed. This may be due to the better quality of the wastewater. All the adsorbed total suspended solids and oil and grease were recovered.

## 4. Conclusions

This work shows that a new generation of silicon carbide membranes can be used to ensure extremely high removals of total suspended solids and oil and grease and moderate removals of chemical oxygen demand (COD) and total organic carbon (TOC) from olive mill wastewaters. Removal of oil and grease was largely due to adsorption/deposition of the compounds on the surface of the membrane and harder to remove using the flux maintenance strategies compared with other fouling agents. 

The employment of backpulses every 10 min is an effective strategy to achieve a reduction of the fouling formation at the surface of the membrane since it enables a release of the adsorbed compounds. When the backpulses are combined with backwashes, the percent of adsorption/deposition of the analysed compounds is further reduced. The combination of backpulses every 10 min and backwash every 1 h helps minimize fouling, maintain flux and avoid high TMP increase. A high reduction of adsorption/deposition of oil and grease in the membrane surface was observed. This result can explain the decrease of the resistance due to the fouling observed when working under the determined optimum constant permeate flux (67 L·m^−2^·h^−1^) together with backpulses every 10 min and backwashes every hour, indicating that oil and grease is an important component of fouling.

To recover the membrane permeability, the simplest and most effective strategy is to rinse and alternate a basic and an acid cleaning solution. All these steps must be performed at controlled temperature, between 60 and 65 °C. Rinsing at 60–65 °C seems to be the step that most contributes to the removal of oil and grease and total suspended solids, followed by the basic cleaning with 4% NaOH.

Results demonstrate that membrane filtration using this new generation of silicon carbide membranes is extremely effective to remove total suspended solids and oil and grease from different real olive mill effluents and thus constitute a promising alternative to conventional wastewater treatment processes.

This process allowed us to obtain water with concentrations of total suspended solids and oil and grease below the maximum levels legislated for direct discharge in the environment. However, high contents of dissolved organic components are still present and must be further removed. Processes such as nanofiltration [39] or advanced oxidation processes [2,7,49] may be good options to reduce it and to guarantee the production of high quality water.

## Figures and Tables

**Table 1:** Characteristics of the silicon carbide (SiC) membrane module used.

**Configuration**	Cylindrical with round channels
**Element Dimensions (mm)**	Length: 305 ± 1; Diameter: 25 ± 1
**Area (m^2^)**	0.09
**Number of Channels**	31
**Channel Diameter (mm)**	3
**Manufacture Nominal Pore Size (µm)**	0.04
**Maximum Operation Pressure (bar)**	10
**Temperature Tolerance (°C)**	Up to 800
**pH Tolerance**	0–14
**Hydraulic Permeability (Experimentally Determined) (L·m^−2^ h^−1^·bar^−1^) ^a^**	2083 ± 127
**Porosity (%) ^a,b^**	2.7 ± 0.2
**First contact angle (°) ^c^**	19 ± 4
**Velocity of decreasing contact angle (°s^−1^) ^c^**	35 ± 6

a: reference [33]; b: average of the values determined in two zones of the membrane; c: average of the values determined in nine zones of the membrane.

**Table 2:** Characterization of the real olive mill wastewater samples collected and limits imposed by legislation.

Parameter	Average Concentration	Portuguese Legislation (DL 236/98) Concentration	European Legislation (91/271/EEC) Concentration
Total solids (mg/L)	6260 ± 770	n.d.	n.d.
Total suspended solids (mg/L)	2010 ± 1105	60	35
COD (mg O_2_/L)	8720 ± 1148	150	125
TOC (mg/L)	2555 ± 301	n.d	n.d.
Oil and grease (mg/L)	275 ± 60	15	n.d.

n.d.: not defined.

**Table 3:** Permeate flux and flux maintenance strategies applied in the different filtration tests.

Conditions	Test 1	Test 2	Test 3	Test 4
Imposed constant permeate flux (L·m^−2^·h^−1^)	67	67	67	67
Flux maintenance strategy	No	Backpulse each 10 min (duration: 0.8 s; TMP = −3 bar)	Backpulse each 10 min + Backwash each 2h (duration: 2 s; Jb = 1 m^3^·h^−1^·m^−2^)	Backpulse each 10 min + Backwash each 1 h (duration: 2 s; Jb = m^3^·h^−1^·m^−2^)

**Table 4:** Comparison of ∆*TMP* and effectiveness (𝝶) of backpulses (test 2) and backwashings (test 3 and test 4) as flux maintenance strategies.

	Test 1	Test 2	Test 3	Test 4
*ΔTMP_test_*	0.53	0.48	0.43	0.28
𝝶	0%	9%	19%	47%

**Table 5:** Percent total rejection and adsorption/deposition of total solids, total suspended solids, chemical oxygen demand (COD), total organic carbon (TOC) and oil and grease.

Parameter	Test 1		Test 2		Test 3		Test 4	
	% Rejection	% Ads/Dep	% Rejection	% Ads/Dep	% Rejection	% Ads/Dep	% Rejection	% Ads/Dep
Total solids	37	12	29	2	49	0	56	12
Total suspended solids	>99.9	49	>99.9	24	99	19	>99.9	22
COD	57	30	37	0	64	1	69	3
TOC	49	26	60	23	68	0	64	0
Oil and grease	97	89	97	76	99	74	99	46

**Table 6:** Characterization of feed and permeate in terms of total solids, total suspended solids (TSS), chemical oxygen demand (COD), total organic carbon (TOC) and oil and grease in tests 1–4.

Concentration (mg/L)	Test 1			Test 2			Test 3			Test 4		
	Feed 0 h	Feed 24 h	Permeate 24 h	Feed 0 h	Feed 24 h	Permeate 24 h	Feed 0 h	Feed 24 h	Permeate 24 h	Feed 0 h	Feed 24 h	Permeate 24 h
Total solids	7012	5664	4416	5232	4692	3728	6148	6072	3108	6644	5168	2944
TSS	1525	770	1.7	843	640	1.8	2233	1813	12	3432	2460	5.6
COD	8824	5752	3756	7085	6715	4465	9708	9264	3516	9264	8468	2832
TOC	2247	1530	1152	2812	2031	1120	2813	3126	904	2346	2623	856
Oil and grease	270	30	7.6	250	58	8.5	360	93	4	220	89	3

**Table 7:** Characterization of the olive mill wastewater used in the concentration test in terms of total solids, total suspended solids, chemical oxygen demand (COD), total organic carbon (TOC) and oil and grease.

Parameter	Concentration (mg/L)
Total solids	1946
Total suspended solids	438
COD	1850
TOC	305
